# Infliximab in the treatment of refractory uveitis. Does dose really matter?

**DOI:** 10.1186/1546-0096-9-S1-P219

**Published:** 2011-09-14

**Authors:** MD Sukesh Sukumaran, MD Katherine Marzan, MD Andreas Reiff

**Affiliations:** 1 Division of Pediatric Rheumatology, Children’s Hospital Los Angeles, Los Angeles, CA, USA

## Background

Infliximab has been shown to be beneficial in treating pediatric uveitis. No data exist on optimal dosing and true efficacy of higher dosing.

## Objectives

To compare the efficacy of low dose (LD) infliximab (<10mg/kg), moderate dose (MD) infliximab (≥10-15mg/kg) and high dose (HD) (≥15-20mg/kg) in the treatment of various forms of uveitis.

## Methods

We conducted a retrospective study at Children’s Hospital Los Angeles and Miller Children’s Hospital, Los Angeles. We performed comprehensive medical record review to identify demographic information and clinical data. Ocular outcome was assessed by anterior chamber cell density, flare, visual acuity, intra ocular pressure and ability to reduce or stop concomitant topical or systemic steroids.

## Results

The mean age of patients was 11.2 years and mean duration of uveitis was 33 months. All patients had bilateral eye disease. 71% of children had preexisting complications including glaucoma and cataracts. Of the 34 patients, there were 6 (18%), 19 (56%) and 9 (26%) patients in LD, MD and HD group respectively.

Characteristics, duration of uveitis and response to therapy are described (Table 1).

**Table 1:** 

	LD	MD	HD
Anatomic classification of Uveitis among cohort (%)			

Anterior Uveitis	100	31	33

Intermediate Uveitis	0	<1	1

Posterior Uveitis	0	26	33

Panuveitis	0	42	44

Mean age at diagnosis of uveitis (yrs)	8.9	8.4	8.5

Mean age of onset of treatment (yrs)	10.8	10.9	12.1

Mean duration of other medication regimens prior to infliximab (yrs)	2.0	2.6	3.5

Duration of treatment with topical steroids prior to infliximab (mo)	10.6	12.3	15.4

Time to discontinuation of steroids after infliximab (mo)	3.1	9.5	10.2

Patients requiring dose escalation (%)	67	35	55

Average time to dose escalation (mo)	5.1	7.5	14

## Conclusion

We found that treatment with infliximab is beneficial and that dose escalation up to 4 times above the approved dose is often necessary to achieve disease control. LD infliximab was found not to be sufficient in disease control. Although we found MD to be effective, one third of patients in this group required escalation for better disease control. Despite escalated doses, treatment appeared safe in the short term and no adverse events were observed.

